# Assessment of Urban Agglomeration Ecological Sustainability and Identification of Influencing Factors: Based on the 3DEF Model and the Random Forest

**DOI:** 10.3390/ijerph20010422

**Published:** 2022-12-27

**Authors:** Zhigang Li, Jie Yang, Jialong Zhong, Dong Zhang

**Affiliations:** 1 College of Management Science, Chengdu University of Technology, Chengdu 610059, China; 2 The Engineering & Technical College of Chengdu University of Technology, Leshan 614000, China

**Keywords:** 3DEF model, random forest, ecological sustainability, factor discrimination

## Abstract

The evaluation of ecological sustainability is significant for high-quality urban development and scientific management and regulation. Taking the Chengdu urban agglomeration (CUA) as the research object, this paper combined the three-dimensional ecological footprint model (3DEF) and random forest to evaluate the ecological sustainability of the study area and identify the influencing factors. The study results indicate that: (1) From 2000 to 2019, the ecological sustainability of Chengdu urban agglomeration was divided into four types, and the overall ecological sustainability of this region showed a downward trend. The areas with higher ecological sustainability were mainly distributed in the northern part of the urban agglomeration (Mianyang City) and the southern part (Leshan City and Ya’an City), while the cities in the central region (Chengdu City, Meishan City, and Ziyang City) had lower ecological sustainability. (2) The main factors affecting the ecological sustainability of urban agglomerations are industrial wastewater discharge, industrial smoke (powder) dust discharge, and green coverage of built-up areas, followed by urbanization and population size. Through this study, we have two meaningful findings: (a) Our research method in this paper provides a new way to study the factors affecting the ecological sustainability of urban agglomerations. (b) The results of the identification of influencing factors might be the reference for urban environmental infrastructure construction and urban planning.

## 1. Introduction

Since the reform and opening up, China’s economy has developed rapidly. However, many ecological and environmental problems have emerged. China’s environmental protection and sustainable development are full of challenges [1]. Among the Sustainable Development Goals (SDGs), the ecological Sustainable Development Goals emphasize limiting human activities to what nature can afford. Only by effectively utilizing natural resources and maintaining the coordinated development of the natural environment and social economy can the goal of ecologically sustainable development be realized [2].

The ecological footprint concept was first proposed in 1992 by Ree [3], and then Wackemagel developed it into the ecological footprint model [4]. The model quantifies the consumption and occupation of natural capital by human activities in biologically productive land area and directly expresses the occupation degree of natural capital by human beings [5,6]. Therefore, it is very effective to evaluate ecological sustainability based on this model [7]. However, traditional ecological footprint models only calculate the size of natural capital flow without separating capital flow and capital stock, which cannot reflect the role of natural capital stock on ecological balance [8]. So, Niccolucci [9] introduced the ecological footprint size (*EF_size_*) and ecological footprint depth (*EF_depth_*) to construct a three-dimensional ecological footprint model (3DEF), and the spatial and temporal variation characteristics of global ecological footprint size and depth from 1961 to 2006 were analyzed using this model [10]. The 3DEF model was introduced by Kai Fang for the first time to analyze the capital utilization patterns of Chinese provinces [11]. Fang [12] comprehensively interpreted the *EF_size_* and the *EF_depth_* and the results showed that we could analyze whether human consumption is overloaded from both horizontal and vertical perspectives by using the 3DEF model. The model could be a new method to study ecological sustainable development. Then, Fang [13] analyzed the spatial variation characteristics of natural capital utilization in G20 countries from 1999 to 2008 and found that the traditional three-dimensional ecological footprint model has the problem of offsetting the regional deficit. To overcome this problem, Fang [14] modified the model to obtain a revised 3DEF model and used it to study the characteristics of natural capital utilization in 11 countries. 

Currently, the 3DEF model has been widely used to evaluate the ecological sustainability of different scale study areas, such as the national scale, the provincial scale, and the urban agglomeration scale. On the national scale, Fang [13] found that resource-rich countries have higher *EF_size_* and lower *EF_depth_*; that is, they have higher capital flow and lower capital stock, and the prospect of ecological sustainable development is limited by resource endowment and the economic development level. In addition, renewable resources might restrict the utilization efficiency of natural capital flow, and there is a significant negative correlation between the consumption of natural capital stock and the level of social and economic development [14]. On the provincial scale, Wang et al. [15] measured the three-dimensional ecological footprint of 12 cities in Inner Mongolia from 2010 to 2016 and examined the driving factors. The results showed that there was over-utilization of resources. The determinants of ecological surplus/deficit were not only the natural endowment but also population density, industrial structure, and the technological level. Based on the revised 3DEF model, Wu [16] analyzed the sustainability and decoupling of natural capital utilization in 30 provinces of China from the two dimensions of capital flow and stock and believed that increasing footprint scale and decreasing footprint depth are conducive to ecologically sustainable development. On the urban agglomeration scale, in Du’s research [17], the *EF_depth_* was influenced by the quantity and structure of energy consumption and had an inverted “N”-shaped relationship with economic development. Wang et al. [18] conducted PLS modeling with the *EF_depth_* as the explained variable and socioeconomic indicators as the explanatory variables and found that the natural capital utilization patterns of the Guangdong–Hong Kong–Macao Greater Bay Area could be divided into four categories. Ecological sustainability was best when capital stock consumption was reduced and capital flow was abundant. Additionally, Yang et al. [19] and Chen et al. [20] showed that evaluating the ecological sustainability of urban agglomerations by the 3DEF model is reliable. 

Overall, the use of the 3DEF model to evaluate ecological sustainability has been quite mature. However, due to the nonlinear relationship between ecological footprint indicators and socioeconomic indicators [21], the high number of features, and the small sample sizes [22], there are few studies on the influencing factors of ecological sustainability from the perspective of data mining. Fortunately, the random forest method is not dependent on the sample size, and the OOB algorithm with the importance of measure variables extracts multiple samples from the original sample through the bootstrap resampling method [23], which is a data mining method that can overcome the nonlinear relationship and poor data information [24]. 

With the Chengdu urban agglomeration (CUA) as an example, this work is a preliminary attempt to remedy these gaps by investigating ecological sustainability from the standpoint of machine learning (ML). As a starting point, we used the 3DEF model to determine the *EF_size_* and *EF_depth_* of the study region. Using the two indicators, the study area’s ecologically sustainable types can then be segmented. The random forest technique was finally established to pinpoint the variables that affect ecological sustainability. The following are some possible innovations from this study: (1) The factors affecting ecological sustainability were initially discussed using footprint methods and machine learning; (2) our research methodology in this paper offers a new way to investigate the factors affecting the ecological sustainability of urban agglomeration; and (3) our study may serve as a guide for the analysis of ecological footprint indicators in the case of small samples.

## 2. Materials and Methods

### 2.1. Study Area

The Chengdu urban agglomeration (CUA) is located in western China. It includes eight cities, with Chengdu as the center. The overall terrain of the CUA is flat, dominated by plains, basins, and hills. As the core area of Sichuan Province’s multi-point and multi-polar support development strategy, it plays a vital role in the economic development of Sichuan Province. At the end of 2020, the regional land area was about 78,000 square kilometers, with a permanent population of 38.518 million and a GDP of RMB 2829.56 billion.

### 2.2. Data Sources

This study uses the Chengdu urban agglomeration as the research object and collected socioeconomic and land use data in 4 years (2000, 2010, 2015, and 2019). Among them, the socioeconomic data comes from the statistical yearbooks of various cities, and the land use data and administrative division vector data come from the Resource Environment and Science Data Center of the Institute of Geographical Sciences (https://www.resdc.cn/Default.aspx, accessed on 8 July 2022). In the ecological footprint calculation, the equilibrium and yield factors were determined by referring to existing research results [25,26], as shown in Table 1. According to the scale characteristics of the study area, the consumption account of biological resources was updated from “global hectare” to “provincial hectare”, that is, the average output of various consumer goods in Sichuan Province. According to The General Rules for Calculation of China’s Comprehensive Energy Consumption (*GB/T2589-2008*), the energy consumption is converted into the area of fossil energy land and building land based on the low calorific value generated per kilogram of fossil fuel, as shown in Table 2. Since economic, social, and environmental factors impact the use of natural capital [16], this paper selects nine elements, as shown in Table 3.

### 2.3. Methods

**Figure 1:**
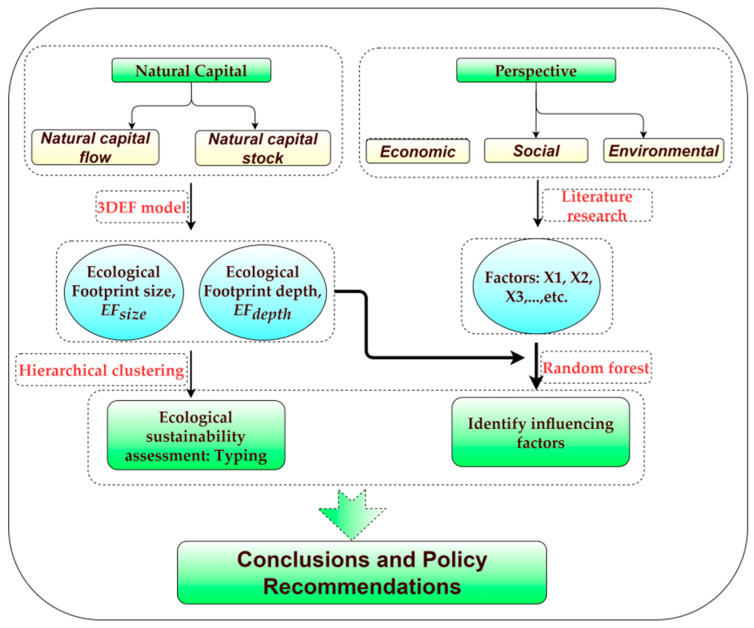
Research framework.

In this study, the improved three-dimensional ecological footprint model was used to determine the ecological footprint size (*EF_size_*), ecological footprint depth (*EF_depth_*), and three-dimensional ecological footprint (EF_3D_) of 8 cities in the Chengdu urban agglomeration in 2000, 2010, 2015, and 2019. First, the *EF_size_* and *EF_depth_* were used to assess the research area’s ecological sustainability. Then, using the random forest OOB algorithm, the Chengdu urban agglomeration’s ecological sustainability impact elements were discovered and examined. The two sections that make up the research framework for this paper are (1) evaluating ecological sustainability and (2) identifying the influencing elements (Figure 1).

#### 2.3.1. Three-Dimensional Ecological Footprint Model

**Figure 2:**
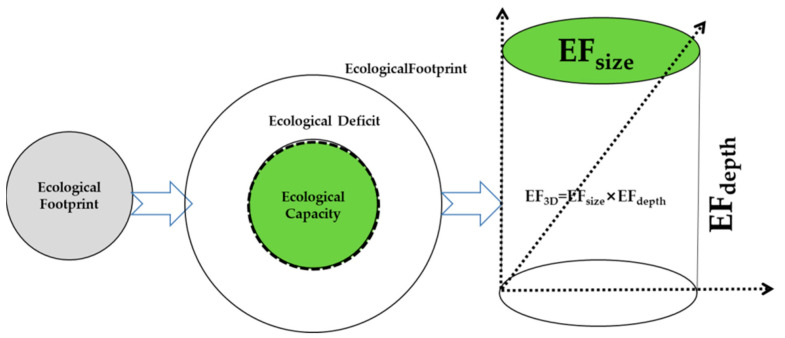
The evolution of ecological footprint model from a one-dimensional model to a three-dimensional model.

The ecological footprint model has experienced the evolution from a one-dimensional ecological footprint to two-dimensional and three-dimensional models (See the Figure 2).

A one-dimensional ecological footprint converts biological resources into the land area to quantify human utilization of natural resources [27]. The calculation formula for one-dimensional ecological footprint is as follows.

(1) $$ef=\sum r_{j}\frac{C_{i}}{Y_{i}}$$

(2) EF=ef×N

where ef is the per capita ecological footprint (hm^2^/cap); EF is the ecological footprint (hm^2^); $r_{j}$ is the equivalence factor; $Y_{i}$ is the average product of item i; $C_{i}$ is the consumption of item i.

A two-dimensional ecological footprint increases the calculation of ecological carrying capacity. The difference between ecological footprint and ecological carrying capacity (i.e., ecological profit and loss) is used to determine whether the ecological footprint can meet the needs of human production activities. The calculation formula for a two-dimensional ecological footprint is as follows.

(3) $$ec=\sum a_{j}\times r_{j}\times y_{j}\times 0.88$$

(4) EC=ec×N

(5) ED=EF−EC

where ec is the per capita ecological carrying capacity (hm^2^/cap); EC is the ecological carrying capacity (hm^2^); ED is the ecological profit and loss (hm^2^); j is the land type; $a_{j}$ is the per capita area of land j; and $y_{j}$ is the yield factor.

The three-dimensional ecological footprint model also introduces two indicators of footprint breadth and depth to quantify the relationship between natural capital stock and flow on a two-dimensional basis [9]. Based on the research of Chinese scholar Fang Kai, a revised three-dimensional ecological footprint model was obtained [14]. The calculation formula for the fixed three-dimensional ecological footprint is as follows.

(6) $$EF_{size}=\sum \min\{EF_{i},EC_{i}\}$$

(7) $$EF_{depth}=1+\frac{ED_{i}}{EC_{i}}=1+\frac{\sum \max\{EF_{i}-EC_{i},0\}}{\sum EC_{i}}$$

(8) $$EF_{3D}=EF_{size}\times EF_{depth}$$

where $EF_{size}$ is the ecological footprint size, which indicates the size of natural capital flow; $EF_{depth}$ is the depth of ecological footprint, which represents the size of natural capital stock; and $EF_{3D}$ is the three-dimensional ecological footprint. Obviously, in the Equation (7), when the $EF_{i}\leq EC_{i}$, the $EF_{depth}$ is the original length “1”, which means the capital flow is surplus.

#### 2.3.2. The OOB Algorithm in Random Forest

Random forest is a statistical learning theory. It uses the bootstrap re-sampling method to extract multiple samples from the original samples, conducts decision tree modeling for each bootstrap sample, and then combines the predictions of numerous decision trees to obtain the final prediction result through voting [28,29]. The random forest can be well used to evaluate the importance of variables [30] and is widely used in ecology [31].

There are usually Gini importance and mean square error reduction methods for variable importance measurement using random forest, among which the calculation steps of the mean square error reduction method are as follows.

Step 1: Calculate the mean square error (*MSE*) of each decision tree’s out-of-bag data (*OOB*), and the calculation formula is as follows.

(9) $$MSE_{t}=\frac{1}{N_{t}}{\sum_{i=1}^{N_{t}}\left(y_{i}-{\hat{y}}_{i,t}\right)}^{2}$$

Step 2: Replace the target variables randomly and calculate the new mean square deviation. The calculation formula is as follows.

(10) $$MSE_{t}(\nu )=\frac{1}{N_{t}}{\sum_{i=1}^{N_{t}}\left(y_{i}-{\hat{y}}_{i,t}(\nu )\right)}^{2}$$

Step 3: The importance measure of variables was calculated based on the mean square deviation before and after replacement, and the calculation formula is as follows:

(11) $$VI(\nu )=MSE(\nu )=\frac{1}{n}\sum_{t=1}^{n}\left(MSE_{t}-MSE_{t}(\nu )\right)$$

(12) $$R(\nu )=\frac{VI\left(\nu \right)}{\sum VI\left(\nu \right)}$$

where in the Equations (9)–(12), $N_{t}$ is the number of cities in the tree with OOB data; ${\hat{y}}_{i,t}$ is the predicted value of the dependent variable of the city under the tree; ${\hat{y}}_{i,t}(\nu )$ is the predicted value of the dependent variable of the city under the new tree after random substitution of variables; VI(ν) is the importance of variables; and R(ν) is the variable importance ratio.

#### 2.3.3. A Combination of the Two Approaches

Ecological footprint size (*EF_size_*) and depth (*EF_depth_*) of the study area can be obtained through the three-dimensional ecological footprint model (Equations (1)–(7)). These two variables are taken as explained variables, and the indicators (*X*1, …, *X*9 in Table 3) representing the three aspects of economy, society, and environment are taken as explanatory variables. Then, we conducted random forest regression model fitting to investigate the explanatory ability of indicators and then calculated the importance proportion of all variables (*X*1, …, *X*9) based on the OOB algorithm (see “Section 2.3.2”) so as to complete the task of identifying influencing factors.

## 3. Results

### 3.1. Ecological Footprint Size

**Figure 3:**
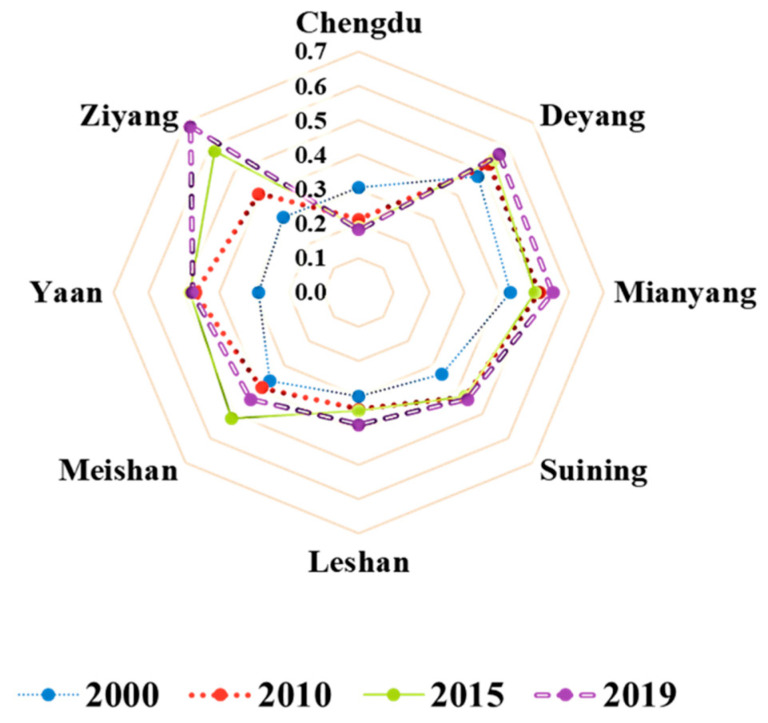
The *EF_size_* of the eight cities from 2000 to 2019 (hm^2^).

The ecological footprint size represents the human occupation of natural capital flow. As shown in Figure 3, the per capita *EF_size_* of each city showed an overall increasing trend over time, but the value of per capita *EF_size_* varies significantly among cities. In 2019, the natural capital flows of Ziyang City, Deyang City, and Mianyang City were more fully occupied, with per capita *EF_size_* exceeding 0.5 hm^2^. It is worth noting that the per capita *EF_size_* of Ziyang City changed the most from 2000 to 2019, while the other seven cities had little change, and the per capita *EF_size_* values for Chengdu City and Leshan City were always within the range of 0.2–0.4 hm^2^.

**Figure 4:**
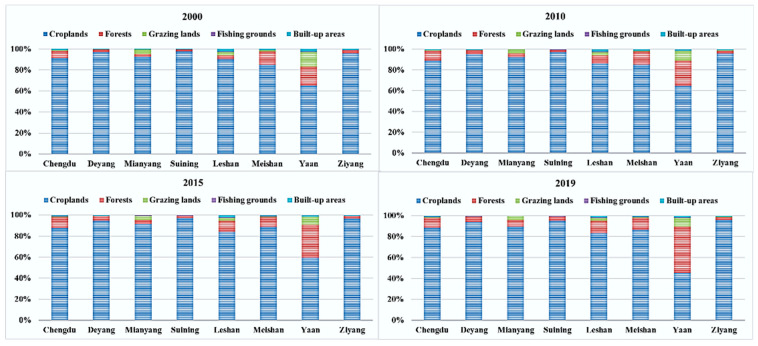
The *EF_depth_* of land types composition from 2000 to 2019.

Further analysis of the *EF_size_* of each land type (as shown in Figure 4) found that the *EF_size_* ratio of cultivated land in all cities was the largest, and the *EF_size_* ratio of cultivated land in other cities is more than 90%, except Ya’an City. It means that the regional development of the CUA is highly dependent on cultivated land. However, the proportion of *EF_size_* in forests, grazing lands, fishing grounds, and built-up areas varies among cities. Except for the *EF_size_* proportion of forests in Ya’an City increasing significantly, the *EF_size_* proportion of different land types remained relatively stable from the perspective of time until 2019. Therefore, the *EF_size_* of different kinds of land in each city in 2019 can be analyzed in detail. In 2019, the proportion of the *EF_size_* of croplands was the largest, with the highest being Ziyang City (96.09%) and the lowest being Ya’an City (45.41%). It is followed by forest *EF_size_* (Ya’an City is the highest at 43.99%, and Ziyang City is the lowest at 2.64%), grazing lands (Ya’an City is the highest at 8.59%, and Ziyang City is the lowest with 0.11%), built-up areas (Leshan City is the highest with 1.74%*,* and Mianyang City is the lowest with 0.35%), fishing grounds (Leshan City is the highest with 0.57%, and Deyang City is the lowest with 0.15%). In general, these four land types have little influence on the ecological footprint size of the CUA.

### 3.2. Ecological Footprint Depth

**Figure 5:**
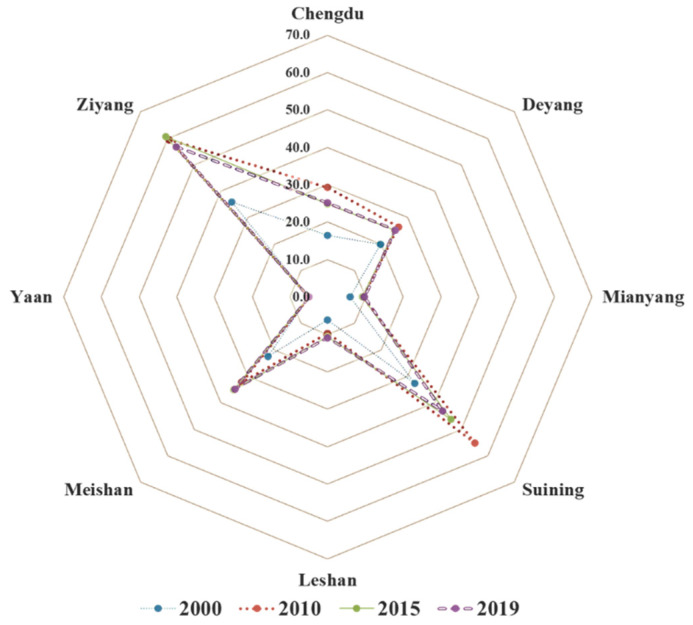
The *EF_depth_* of eight cities from 2000 to 2019.

The *EF_depth_* represents the consumption of the natural capital stock. Only Ya’an’s *EF_depth_* is always at the original length, while the other cities exceed the actual length (Figure 5), which means that only Ya’an’s natural capital flow can meet the regional development needs. Meanwhile, much of the natural capital stock has been consumed in the remaining seven cities. There are apparent differences in *EF_depth_* values among cities. Specifically, the *EF_depth_* of Mianyang is lower than 10, and the value of Leshan’s *EF_depth_* is between 6 and 11. The *EF_depth_* values in Deyang and Chengdu ranged from 16 to 26, and Meishan and Suining were between 30 and 50. Ziyang had the highest *EF_depth_* at 61. The difference in *EF_depth_* indicates the difference in capital stock consumption and unsustainable development level in different cities. In terms of time, the development trend of *EF_depth_* in the eight cities is also different. The average annual change of *EF_depth_* showed an increasing trend of seven cities excluding Ya’an. The average annual growth rates were as follows: Leshan (6.60%), Mianyang (5.79%), Ziyang (5.04%), Meishan (5.04%), Chengdu (4.85%), Suining (3.08%), and Deyang (2.73%).

Table 4 shows the *EF_depth_* exploration of five land types (excluding carbon capture land) in 2000 and 2019. The *EF_depth_* of croplands, built-up areas, and forests (excluding Meishan Ziyang, Deyang, and Chengdu) remains 1, which shows that under the condition of the overall urban ecological deficit, the forests and built-up areas have an ecological surplus and are in a sustainable development state. Combined with the growth of the built-up areas in each city, it shows that the expansion of built-up areas and the strengthening of industrialization in urbanization can alleviate the ecological pressure of built-up areas to a certain extent. However, the *EF_depth_* of other land types showed overdevelopment, and the difference in the *EF_depth_* of grazing land was the largest. The variation coefficient of *EF_depth_* of grazing land in 8 cities increased from 88.79% to 94.3% (From 2000 to 2019), which indicates that the consumption of natural capital stock of grazing land is large and different among cities.

### 3.3. Three-Dimensional Ecological Footprint

The results of the three-dimensional ecological footprint of the CUA in 4 years are shown in Table 5. Specifically, only Chengdu presents a downward trend, declining by 8.32%. On the contrary, the other seven cities have a more substantial increase, as follows: Ziyang (251.77%), Leshan (126.56%), Mianyang (113.08%), Meishan (88.82%), Suining (72.74%), Ya’an (66.56%), and Deyang (50.81%). Therefore, we can infer that the characteristics of comprehensive utilization of regional resources in Chengdu urban agglomeration are as follows: Chengdu is the core of the radial development, and Chengdu’s industrial migration makes the surrounding cities develop rapidly. Furthermore, especially in 2016, Chengdu incorporated Jianyang city, an area under the jurisdiction of Ziyang city, into its administrative region.

### 3.4. Classification of Urban Agglomeration Ecological Sustainability

**Figure 6:**
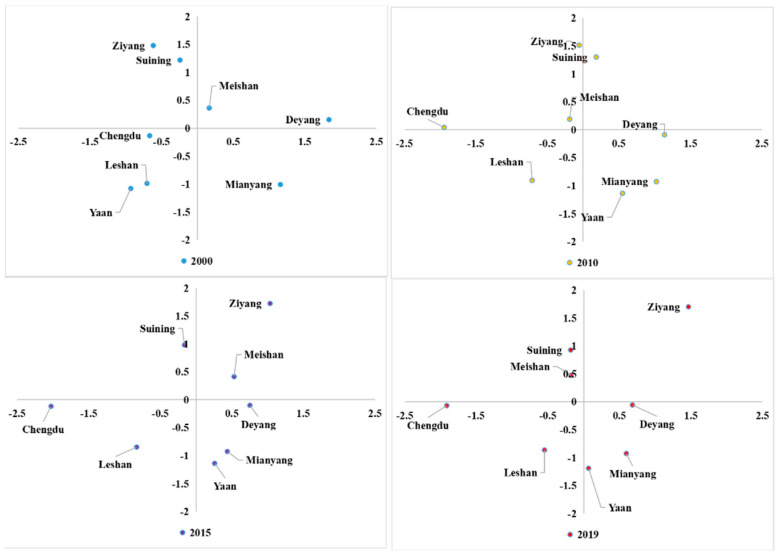
Quadrant scatter plots of the relationship between regional footprint depth and regional footprint size of each city.

According to the standardized size relationship of the *EF_size_* and *EF_depth_* (Figure 6) and the systematic clustering results of standardized data, the ecological sustainability of 8 cities in the study area was intuitively divided into four types: 

Type 1: both the *EF_size_* and *EF_depth_* are high, indicating high natural capital utilization and massive capital stock consumption. Cities in this category have the most extraordinary ecological environment pressure and the lowest degree of ecological sustainability. 

Type 2: the *EF_size_* is low and the *EF_depth_* is moderate, manifested by the reasonable utilization of natural capital and the consumption rate of capital stock is higher than that of capital flow. These cities are facing more significant pressure from regional development and have a low degree of ecological sustainability. 

Type 3: both the *EF_size_* and *EF_depth_* are moderate, which shows that the utilization rate of natural capital flow is higher than the utilization rate of the stock. These cities have high ecological sustainability 

Type 4: the *EF_size_* is moderate, and the *EF_depth_* is low, manifested by lagging utilization of natural capital stock and dominated by capital flow utilization, these cities have the highest ecological sustainability.

**Figure 7:**
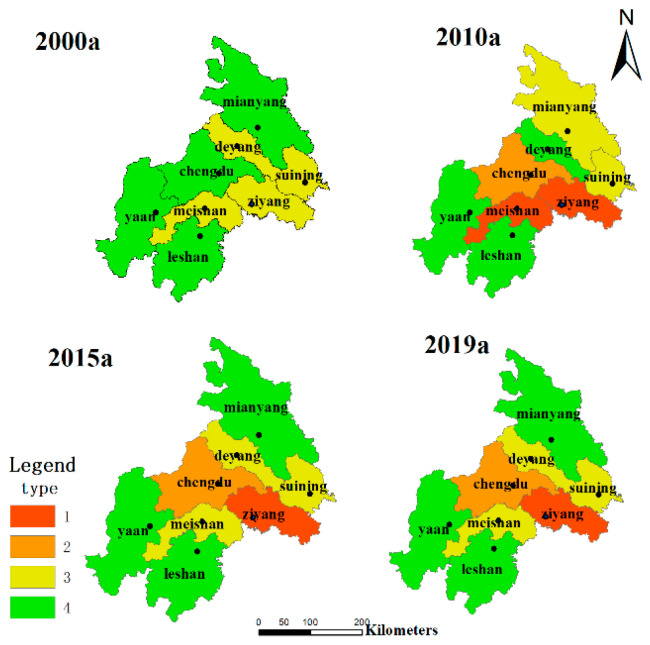
Spatial and temporal distribution of ecological sustainability in 8 cities.

As shown in Figure 7, obviously, during the study period (2000–2019), the ecological sustainability of the Chengdu urban agglomeration became worse, and some cities’ natural capital utilization types also changed significantly. For example, Chengdu changed from type 4 to type 2, and Ziyang changed from type 3 to type 1. The intensification of capital stock consumption led to a downward trend of regional ecological sustainability [32]. Furthermore, regarding geographical location, ecological sustainability is low, mainly concentrated in the central cities (such as Chengdu and Ziyang). In contrast, ecological sustainability is high in the north and south (in cities such as Mianyang, Ya’an, and Leshan). The spatial distribution characteristics of resource endowments in this region are consistent [33].

### 3.5. Identification Results of Influencing Factors

The nine indicators related to economy, society, and environment are selected (*X*1, *X*2, …, *X*9) as explanatory variables. Natural logarithms of the *EF_size_* and the *EF_depth_* were used as explained variables for random forest regression model fitting and the importance ratio (Table 6). Table 6 shows the OOB measurement results of 2000, 2010, 2015, 2019, the four years as a whole, and the corresponding goodness-of-fit R^2^ of the model. First, all R^2^ values are more significant than 0.7, indicating that all models are valid. Second, for footprint breadth, the importance of economic and social indicators for the *EF_size_* increased from 2000 to 2019. The volume of economic indicators rose from 16.0% to 24.2%, and the importance of social indicators increased from 19.4% to 37.4%. However, the importance of environmental indicators decreased from 64.4% to 38.4%. Third, for the *EF_depth_*, the situation changed significantly. The volume of environmental indicators has increased dramatically, from 40.4 percent in 2000 to 72.9 percent in 2019. On the other hand, the importance of economic and social indicators decreased significantly. Fourthly, according to the importance ratio of the accurate data, the three indexes that have the most significant impact on the *EF_size_* are *X*6 (21.8%), *X*9 (20.3%), and *X*4 (15.4%), with a cumulative ratio of more than 50%. The three indexes that have the most significant influence on the *EF_depth_* are *X*8 (35.8%), *X*9 (13.4%), and *X*3 (14.0%), which are the three indexes with the most significant importance, and the accumulative proportion exceeds 60%. It can be seen that, on the whole, environmental factors in the study area have the most significant impact on ecological sustainability.

## 4. Discussion

### 4.1. Influence the Effectiveness and Universality of Factor Identification Method

#### 4.1.1. The Effectiveness of the Method

From the point of view of data characteristics, the *EF_size_* and the *EF_depth_* measured by the 3DEF model are numerical values with exponential properties, and there is a complex nonlinear relationship between a series of indicators of the economy, society, and environment, so the traditional factor identification method is invalid [34]. Therefore, previous studies just used the 3DEF model to measure ecological sustainability indicators [35] and conducted a simple descriptive statistical analysis of the measurement results [17]. The random forest can overcome the nonlinear relationship between variables [36]. It is meaningful to combine the measurement results of 3DEF model with the random forest. Fortunately, the R^2^ of the model obtained in Table 6 is very high, ranging from 0.75 to 0.90, indicating that this algorithm is indeed feasible.

#### 4.1.2. The Universality of the Method

Computing the loss reduction on the out-of-bag (OOB) instead of the in-bag training samples makes the variable importance measurement unbiased [37]. So, the value of R^2^ increases gradually with time, implying that the random forest algorithm selected in this paper successfully identifies the factors affecting the ecological sustainability of urban agglomeration and has extensibility. Moreover, due to the extremely high ecological fragility of the Chengdu urban agglomeration, environmental protection is the primary measure to maintain and improve ecological sustainability [38,39]. That is to say, the realistic results also confirm the rationality of our method. In addition, whether the random forest is used to determine the key attributes of the fishery improvement projects (FIPs) [40] or obtain the importance measure of single and group variables, it has achieved good results [41]. In particular, the random forest has universal applicability to urban environmental problems, for example, the study of urban impervious surface extraction [42], research on the impact of spatial scale on urban ecological environment and human activities [43], and so on.

### 4.2. The Main Factors Affecting Ecological Sustainability

Among the three influencing factors of economy, society, and environment, the environmental factor has become the main influencing factor of ecological sustainability. The social and economic factors have more and more influence on utilizing natural capital flow. The research of this paper can provide the following two aspects of exploring the ecologically sustainable development and management of urban agglomeration. First, managers should focus on improving the utilization efficiency of natural capital flow to enhance the ecological sustainability of urban agglomeration. Industrial wastewater discharge (*X*6) and green coverage of built-up areas (*X*9) are significant to the *EF_size_*. The water environment resources are an essential source of natural capital flow. Reducing industrial wastewater discharge in cities is conducive to protecting water environment resources, and the utilization efficiency of water resources will be improved [44]. Land resources are also essential donors of natural capital flow.

The improvement of green coverage in built-up areas can enhance the resilience of the urban ecological environment and the efficiency of land use [45]. With the acceleration of urbanization, human demand for natural capital increases, even if the utilization efficiency of natural capital flow is improved. Because the acceleration of urbanization makes consumption of the capital stock faster, urban ecological sustainability still faces a severe test [46]. Second, we could explore measures to balance the accumulation of natural capital and stock consumption to promote the ecological sustainability of urban agglomeration. Industrial smoke and dust emission (*X*8) and green coverage of built-up areas (*X*9) are the main influencing factors of the *EF_depth_*. If we reduce emissions of pollutants, we can increase the accumulation of natural capital and thus increase the consumptive capacity of the capital stock [47]. Urban green space promotes ecosystem supply and regulation services [48]. The surge in population size in urban agglomerations brings a particular burden to ecological carrying capacity [49,50]. Therefore, maintaining the appropriateness of urban population size can alleviate the consumption of natural capital stock [51]. A suitable factor identification method is the cornerstone of a comprehensive evaluation study [52], and the research method in this paper may provide a basis for constructing an evaluation system for regional sustainable development research.

### 4.3. Limitations and Extensions of this Study

The study has several limitations. First, there are limitations to land use data used for ecological footprint accounting, such as low spatial and temporal resolution of data or high costs and long periods [53]. In fact, in future research, we may combine remote sensing interpretation, the Land-Parcel Identification System (LPIS), and the original Public Land Survey (PLS) in small areas to obtain high-accuracy data. Second, in the selection of indicators, the method of literature research has certain coverage limitations. In future studies, statistical methods such as structure equation modeling (SEM) and principal component analysis (PCA) can be combined to make the selection of indicators more comprehensive and objective. Thirdly, we need to further narrow the administrative scale of the research area (for example, take towns or streets as research objects) to make the research conclusions more targeted and the policy suggestions more operable. Finally, this study is the first to combine footprinting and machine learning methods despite some limitations. 

## 5. Conclusions

Taking the Chengdu urban agglomeration as the research object, this paper first calculates the breadth and depth of the ecological footprint of this region by using the three-dimensional ecological footprint model, then divides the ecological sustainability types of the urban agglomeration according to it. Then it introduces the random forest OOB algorithm to identify the influencing factors of ecological sustainability and finally draws the following conclusions:

(1) The ecological sustainability of the study area was divided into four categories.
  (a) The first category, the least ecologically sustainable, is characterized by high utilization of natural capital and massive consumption of capital stock. In this category, cities (such as Ziyang and Meishan in 2019 and Meishan in 2010) face tremendous ecological pressure and unsustainable risks.
  (b) Category two has low ecological sustainability because of the moderate use of natural capital and faster consumption of capital stock than flow, and cities (such as Chengdu in 2010, 2015, and 2019) are facing more significant pressure from regional development. As a result, they have a low degree of ecological sustainability.
  (c) The third category (with cities such as Suining Deyang, and Meishan in 2000, 2015, and 2019) has high ecological sustainability and is characterized by the utilization rate of natural capital flow being higher than the rate of stock consumption.
  (d) The last category (with cities such as Leshan and Ya’an in 2000, 2010, 2015, and 2019) has the highest ecological sustainability, which means the natural capital stock consumption lags, and the capital flow utilization leads.
(2) The environment (the average importance ratio is 58.2%) is the main aspect affecting the ecological sustainability of urban agglomerations, among which the discharge of industrial wastewater, industrial smoke (powder) dust, and the green space of built-up areas are the most important. Therefore, we propose to improve the utilization efficiency of natural capital flow and the consumption resistance of stock by strictly controlling the discharge of industrial waste water and industrial smoke (powder) dust. The improvement of urban ecological sustainability can be used as a reference for evaluating the efficiency of urban environmental management.
(3) Social (the average importance ratio is 25.9%) and economic (the average importance ratio is 15.9%) aspects also impact the ecological sustainability of urban agglomerations, especially on natural capital accumulation and stock consumption. At the same time as accelerating urbanization, maintaining the appropriateness of population size and enlarging urban green space can improve ecological sustainability. Furthermore, it is suggested to strengthen urban infrastructure construction, especially the GI (green infrastructure), to promote high-quality economic development and improve people’s living standards.
(4) The random forest OOB algorithm overcomes the nonlinearity between the ecological footprint index and the economy–society–environment index. The model accuracy is higher than that of a traditional regression model. In the study of environmental assessment and management, the combination of machine learning methods and footprinting methods is feasible and reliable.

## Figures and Tables

**Table 1:** Accounting account of three-dimensional ecological footprint of the CUA.

Account	Sub-Account	Consumer Goods Items	Land Types	Equivalence Factor	Yield Factor
Biological resources	farm products	grains (grains, tubers, beans), oil, vegetables and edible fungi, sugarcane, tobacco	croplands	3.39	1.74
	forest products	garden fruit, tea, cocoons	forests	1.1	0.86
	livestock products	meat (cattle, sheep, pigs, poultry, rabbits), milk (milk, sheep’s milk), eggs	grazing lands	0.56	0.51
	aquatic products	aquatic products (fish, shrimp and crabs, shellfish, algae, etc.)	fishing grounds	0.44	0.74
Energy resources	fossil energy	raw coal, natural gas, gasoline, diesel, coke	carbon capture land	1.1	0
	electricity	electricity	built-up area	3.39	1.74

**Table 2:** Average yield and energy conversion coefficient of “province hectare” in the CUA.

Items	Average Yield (kg/hm^2^)	Items	Average Yield (kg/hm^2^)	Items	Energy Footprint * (GJ/hm^2^)	Convert Coefficient (GJ/ton)
grain	5453.472	tea	804.813	raw coal	55	20.934
oil	2189.756	garden fruit	2459.02	natural gas	93	18.003
vegetables and edible fungi	28,912.615	meat	5592.36	gasoline	93	43.124
sugarcane	46,910.696	milk	494.10	diesel	93	42.705
tobacco	2112.329	eggs	1151.27	coke	55	28.47
cocoons	600	aquatic products	1475.079	electricity	1000	11.84

Note: index data with * was calculated in global hectares.

**Table 3:** Socioeconomic and environmental indicators of the CUA.

Perspective	Indicator and Code	Unit
Economic	Economic development, *X*1	RMB 10,000
	Industrial structure, *X*2	%
Social	Population size, *X*3	10,000 people
	Urbanization, *X*4	%
	Government intervention, *X*5	RMB 10,000
Environmental	Industrial wastewater discharge, *X*6	million tons
	Industrial exhaust emissions, *X*7	billion standard cubic meters
	Industrial smoke (powder) dust emission, *X*8	ton
	Greenery coverage of urban area, *X*9	%

**Table 4:** *EF_depth_* of land types.

City	Croplands		Forests		Grazing Lands		Fishing Grounds		Built-Up Areas	
	2000	2019	2000	2019	2000	2019	2000	2019	2000	2019
Chengdu	1	1	1	2.75	10.91	13.27	2.49	7.12	1	1
Deyang	1	1	1	1.18	14.63	15.88	2.23	6.26	1	1
Mianyang	1	1	1	1	1.09	1.41	1.78	5.34	1	1
Suining	1	1	1	1	28.39	35.86	1.32	4.11	1	1
Leshan	1	1	1	1	2.10	2.80	1.02	5.08	1	1
Meishan	1	1	1.04	3.87	15.84	18.74	3.51	10.23	1	1
Yaan	1	1	1	1	1	1	1	1	1	1
Ziyang	1	1	2.80	9.06	29.12	40.85	1.93	4.93	1	1

**Table 5:** Per capita 3D ecological footprint from 2000 to 2019 (hm^2^).

City	2000	2010	2015	2019	Changes from 2000 to 2019	
Chengdu	4.99	6.24	4.73	4.57	−0.41	−8.32%
Deyang	9.50	13.97	13.71	14.33	4.83	50.81%
Mianyang	2.53	4.78	4.61	5.39	2.86	113.08%
Suining	10.90	23.66	19.77	18.83	7.93	72.74%
Leshan	1.84	3.29	3.65	4.18	2.33	126.56%
Meishan	8.10	12.64	18.17	15.30	7.19	88.82%
Yaan	1.43	2.33	2.41	2.38	0.95	66.56%
Ziyang	11.02	24.10	35.19	38.76	27.74	251.77%

**Table 6:** The importance ratio and model performance of indicators in each year.

Project	Time	Economic		Society			Environment				R^2^
		*X*1	*X*2	*X*3	*X*4	*X*5	*X*6	*X*7	*X*8	*X*9	
for *EF_size_*	2000	10.0%	6.0%	4.1%	11.1%	4.2%	10.2%	9.2%	20.0%	25.2%	0.747
	2010	12.9%	10.8%	10.8%	6.4%	10.1%	7.3%	9.5%	14.5%	17.7%	0.806
	2015	8.4%	17.7%	16.2%	14.7%	11.9%	10.4%	5.6%	5.2%	9.9%	0.899
	2019	11.9%	12.3%	15.4%	12.0%	10.0%	7.1%	12.9%	3.8%	14.6%	0.888
	2000–2019	8.5%	13.1%	8.4%	15.4%	3.2%	21.8%	5.5%	3.9%	20.3%	0.934
for *EF_depth_*	2000	15.2%	11.7%	17.8%	6.8%	8.1%	6.4%	5.0%	6.5%	22.5%	0.728
	2010	14.5%	4.9%	8.4%	19.5%	3.5%	10.8%	13.2%	14.8%	10.4%	0.753
	2015	11.8%	8.4%	4.5%	8.8%	8.5%	6.1%	40.4%	7.6%	3.9%	0.768
	2019	9.2%	9.2%	3.3%	5.4%	9.2%	3.6%	12.8%	40.8%	6.5%	0.838
	2000–2019	5.9%	4.4%	14.0%	5.3%	5.5%	9.3%	6.4%	35.8%	13.4%	0.828

## Data Availability

Not applicable.
